# The postulated innocuity of lifetime exposure to aluminium should be reappraised

**DOI:** 10.3389/fonc.2023.1159899

**Published:** 2023-07-24

**Authors:** Stefano J. Mandriota, André-Pascal Sappino

**Affiliations:** Laboratoire de Cancérogenèse Environnementale, Fondation des Grangettes, Chêne-Bougeries, Switzerland

**Keywords:** aluminium, breast cancer, chromosomal instability (CIN), chemical carcinogenesis, cellular transformation

## Abstract

Because of its chemical versatility and abundance in nature, aluminium is employed in a myriad of frequently used products - including cosmetics and food additives - and applications – drinking water purification procedures being an example. Despite what its widespread use might suggest, aluminium’s harmlessness is a matter of debate in the scientific community. In this article we trace the lines of a growing questioning about the potential mutagenic effects of this metal, due to the data produced over the recent years, and with an eye to the discussions currently underway in this regard between the scientific community, industry, and regulatory bodies.

## Introduction

Aluminium is the most abundant metal and, after oxygen and silicon, the third most abundant element in the Earth’s crust, the layer that makes up the Earth’s surface. Because of its high reactivity with oxygen, aluminium is mainly found in minerals in the form of water-insoluble oxides or silicates. The paucity of free aluminum observed in nature could be one of the reasons why this element is not part of any known physiological process and is therefore not essential for life (1, 2). In an acidic environment, however, aluminium recovers its solubility in water. For this reason, acid rain, a natural phenomenon amplified by industrial emissions, enriches soils with free aluminium. The latter, in turn, is taken up by plants, including those destined to the food chain, such as tea. More directly, humans extract aluminium from its natural sediments and include it in a wide variety of products and applications. Examples include cosmetics (antiperspirants, sunscreens, lipsticks, face masks), certain drugs, most vaccines of traditional formulation, food additives, tobacco, as well as drinking water purification procedures. In antiperspirants, aluminium salts block excretion by sweat glands. In food additives, they have a firming, preserving, anti-caking, and raising function. In some antacid drugs, aluminium - in the hydroxide form - reduces acidity in the stomach. These are only a few examples of the chemical versatility of aluminium. Versatility which, together with its negligible price, abundance, and lack of evidence of a deleterious effect - when used at ordinary concentrations - has encouraged its large-scale use for several decades since approximately the late fifties. During our lifetime we are therefore chronically exposed to aluminium through the digestive, dermic, and respiratory routes. The aluminium we absorb reaches relatively high concentrations in the bone, the liver, and the mammary gland (1, 3).

## Toxicity data

Nothing would probably be clearly wrong with aluminium in the absence of its most prominent toxic effects – in our view, inhibition of root growth in plants growing in acidic soils (4) and dialysis encephalopathy, osteomalacia and microcytic anemia occurring in chronic renal disease (5). In the latter setting, aluminium plasma or serum levels reach values up to several hundred μg/L, compared to 1 to 3 μg/L in the healthy population (5) because of deficient renal clearance, an effect further aggravated by aluminium-containing drugs used to counteract hyperphosphatemia or because of exposure to dialysates prepared with water containing aluminium concentrations above 50μg/L (6). Aluminium associated illnesses observed in this specific setting might suggest that when experienced in more ordinary amounts, aluminium is innocuous. Consistent with this view, studies administering aluminium to the drinking water of rats, under the experimental conditions used, defined a no-observed adverse effect level (NOAEL) at 30 mg/kg/day with respect to neuromuscular development (7), meaning that pathological alterations in the latter parameter can only be observed starting from aluminium concentrations approximately 10’000 times higher than those typically measurable in potable drinking water (around 0.1mg/L). These data do not seem to reassure regarding the amounts of aluminium we are exposed to daily, as witnessed by the numerous publications dealing with potential toxic effects of our daily exposure to aluminium and by the many, regularly updated aluminium safety assessment reports emanating from industries and governmental bodies. Low level, chronic exposure to aluminium has long been suspected of being involved in at least two human pathologies: Alzheimer disease (8) and cancer (3, 9). The former link is not consistently supported by existing epidemiological data (10). With respect to the latter, there is currently no conclusive evidence of aluminium’s involvement in cancer. Such evidence, however, has not been thoroughly searched.

## Is there a link between aluminium and cancer?

The debate on a potential role of aluminium in human carcinogenesis currently mainly concerns breast cancer, due to the presence of high concentrations of aluminium salts in cosmetics frequently applied to the breast area such as antiperspirants and sunscreens. But due to the ubiquitous presence of this metal it could be extended to other cancer types. By affecting 1 in 8 women during her lifetime, and with approximately 2.3 million new cases in 2020, breast cancer is the commonest cancer type in women. Breast cancer incidence has strongly increased in western countries, starting approximately from the end of the sixties (11, 12). With only approximately 15% of the cases being genetically inherited – with germline *BRCA1* or *BRCA2* mutation accounting for an approximate half of them – there is consensus in the relevant literature that breast cancer has a strong environmental component. Key arguments in this regard are studies on migrants from eastern Asia, where the breast cancer incidence is generally lower than in western populations, who develop the same rate of breast cancer as the Americans after having moved to the USA (12) and the observation that specific *BRCA* mutations lead to breast cancer earlier in life in patients born after 1940, compared to those born before that year (13). A study of migrants from eastern Asia to the USA showed that “the increase in breast cancer rates is first seen after 10 years’ residence in the USA, but that the maximum increase is not observed until the descendants of the migrants have been resident for one or two generations” (12–14). The latter observation suggests that chronic exposure to or accumulation over time of unidentified environmental carcinogen(s) could be among the factors involved. Environmental factors promoting breast carcinogenesis remain largely undefined, with currently identified ones - alcohol consumption, obesity, cigarette smoke and hormone exposure - accounting for small fractions of the occurring cases (15).

In a hypothesis paper dating back to 2001 (16) British researcher P. Darbre tried to bring a contribution to this scarcely populated landscape by proposing that underarm cosmetics might contribute to breast cancer incidence in western societies. At a first sight Darbre’s hypothesis might seem unlikely since the healthy skin is, in principle, a powerful barrier against external intruders. In addition, individual chemicals present in underarm cosmetics could be presumed to have passed exhaustive toxicological tests that ensure safety – but this is not always the case.

In its simplest terms, the natural story of a tumor can be seen as an interplay of two main components: a genetic change providing a proliferative advantage in the form of a defective growth or survival control, and a physiological proliferation process giving that change the opportunity to expand. This is illustrated by the tumorigenesis of retinoblastoma, a pediatric tumor type where the underlying genetic change is the inactivation of the of the G1/S cell cycle transition “brake” – namely, the pRb protein – and the physiological proliferative process involved is the one taking place in retinoblasts during eye development (17). In the case of breast cancer, mammary epithelial cell proliferation resulting from estrogen and progesterone activities also drives expansion of cell subpopulations carrying a proliferative advantage, but the cellular alteration landscape is more complex, with a great diversity of mutations and marked tumor heterogeneity across cases. Familial forms of breast cancer rely on inherited mutations in DNA damage repair (DDR) genes such *BRCAs*, *PALB2* or *ATM*. DDR genes are also inactivated (through mutation or epigenetic modifications) in a fraction of sporadic breast cancers (18). These observations indicate that un/misrepaired DNA double strand breaks (DSB) are a key component of breast carcinogenesis. But, if defects in DSB repair play a key role in the initiation or early progression of this cancer type, what is the identity of those compounds that, due to their ability to induce DSB, can reasonably be hypothesized to play a role in breast tumorigenesis? And what could aluminium have to do with breast cancer?

Although aluminium is one of the many chemicals present in antiperspirants, there are several associations speaking in favor of its potential link to breast cancer. First, aluminium was first introduced in commercial antiperspirants at the end of the fifties, thus preceding the abovementioned increase in breast cancer incidence by approximately ten years. Second, the abovementioned increase was accompanied by a topological redistribution, with approximately 85% of breast cancers occurring nowadays in the external parts of the breast, close to the axilla, where antiperspirants are applied on a daily basis. It has been proposed that this area of the breast contains a greater proportion of epithelial tissue – and develops cancer more frequently for this reason. But, even if true, this would not explain the observed redistribution (11). Third, axilla’s skin is particularly thin and permeable. Permeability is likely to be enhanced when shaving practices – resulting in micro wounds - precede cosmetic application and to be further altered by chemicals also present in the underarm cosmetic formulations. Polyethylene glycols (PEGs) for instance, a common component of many cosmetic products, are known to enhance skin permeability (19, 20). Fourth, concentrations of aluminium salts in antiperspirants are particularly high (5-10% w/v in average, i.e. in the 1.85-3.70M range) (21). Fifth, the amounts of aluminium measured by several groups in the mammary gland of women living in industrialized countries is relatively high (in the 0.8-87μM range) compared to serum (0.1- 0.3μM) (3). In human nipple aspirate fluids, for example, aluminium was measured in the 4.87-9.95 μM range by inductively coupled plasma mass spectrometry (ICP‐MS) (22). The last three facts suggest that aluminium measured in the mammary gland of women living in industrialized countries could result at least in part from chronic absorption of aluminium contained in antiperspirants or sunscreens applied to the breast area. Whereas a direct measurement of such “mammary” aluminium from cosmetic use is lacking, studies using a single axillary application of ^26^Al isotope in adult female volunteers (23–26) failed to provide information on this point, since they measured absorbed ^26^Al in urine – thus demonstrating that aluminium is dermally absorbed - but not in the breast or other internal organs. In addition, these studies do not reflect the toxicokinetics of aluminium in a long-term dermal exposure scenario, starting from puberty.

## Epidemiological studies

The few existing epidemiological studies – all retrospective in nature – comparing breast cancer incidence between antiperspirant users *vs* non-users have provided conflicting results. One of them (27) measured the aluminium content of a large subsample of breast cancer cases and controls and found that self-reported underarm cosmetic product (UCP, most of which containing aluminium) use correlates with higher aluminium content in the breast. This study reported a statistically significant association between breast cancer incidence and very frequent (more than once a day) use of UCP for women under the age of 30 (27). Another study (28) reported that frequency of UCP use, in combination with underarm shaving, was associated with a breast cancer diagnosis at an earlier age. The effect was particularly marked in women who started UCP use before the age of 16. Mirick et al. (29) found no association between UCP use and breast cancer risk. The latter study did not collect information on frequency or age of use.

Apart from the specific setting of breast cancer incidence in relation to underarm cosmetic use, existing epidemiological data on aluminium and cancer are minimal and mainly come from the occupational setting. Aluminium production consists of three main phases: mining (extraction of bauxite, its principal ore, from the ground); refining (chemical extraction of alumina (Al_2_O_3_) from bauxite); and smelting (electrolytic reduction of alumina to aluminium). Based on epidemiological data on occupational exposure - workers in aluminium smelters exhibit an increased risk of bladder cancer and, to some extent, of lung cancer - the International Agency for Research on Cancer (IARC) has classified the aluminium smelting phase as “carcinogenic to humans” (Group 1) (30). It has been proposed that polycyclic aromatic hydrocarbons (PAHs) - among the several chemicals intervening in aluminium smelting - could be the carcinogens involved (30). The IARC does not currently classify aluminium itself as a carcinogen. Concerning the mining and refining phases, no increase in cancer frequency has been reported in male workers (31, 32). These phases of aluminium production involve exposure to insoluble forms of the metal (32), which may have lower bioavailability compared to soluble forms.

## Carcinogenicity studies

Existing evidence that aluminium induces cancer in whole animals is scarce (33). The two most frequently cited studies reported increased cancer incidence in white Swiss female (but not male) mice (34) or in Long Evans male (but not female) rats (35) receiving aluminium potassium sulphate 5 ppm in the drinking water for lifetime. These studies, old and insufficiently reported, are difficult to interpret with respect to potential carcinogenic effects of aluminium.

## Cell culture studies

We chronically exposed human or mouse normal mammary epithelial cell models to aluminium – in the form of 99% pure aluminium chloride – concentrations in the range of 10-100μM that is, several thousand times lower than the average amount of aluminium present in commercial antiperspirants, and close to the concentrations measured in the normal mammary gland of women living in industrialized countries (see above). In these experiments, chronic aluminium exposure transformed mammary epithelial cells *in vitro*. Of the cell models used, NMuMG cells transformed *in vitro* by aluminium formed tumors and metastasis in NOD SCID and nude mice; whereas HC11 cells transformed *in vitro* by aluminium formed tumors and metastasis in the immunocompetent and immunocompatible Balb/cByJ strain. In both cases, untreated parallel control cells were not tumorigenic (36–38).

As a next step, we investigated the mechanisms by which aluminium transforms mammary epithelial cells. Similar to recognized carcinogenic metals, aluminium was not detectably mutagenic in the Ames test, that uses bacteria (38). When analyzing mRNA profiles at a time point preceding cellular transformation (four weeks exposure), aluminium had little or no detectable gene expression regulation activity in mammary epithelial cells (36). The latter experiments suggested that aluminium transforms mammary epithelial cells through a mechanism not – or not primarily - involving early alteration of gene expression.

In a recent publication (36) we reported that NMuMG and HC11 cell cultures transformed *in vitro* by chronic aluminium exposure consistently exhibit an increased number of unique chromosomal rearrangements – mainly translocations – compared to the respective controls. Chromosomal instability (CI) occurs in virtually every type of human tumor. It consists of alterations in the structure and number of chromosomes and is associated with increased risk of metastasis and treatment resistance. CI is frequently observed in the first steps of tumorigenesis, where it is believed to facilitate the development of cellular transformation through the occurrence of cellular heterogeneity (39). However, when observed in cells already transformed, CI can represent a consequence, rather than a cause, of cellular transformation. In our experiments, metaphase preparations from HC11 cells exposed to aluminium for 24 hours revealed an increase of chromosomal DSBs, an alteration leading to chromosomal translocations if not repaired properly. These results provide a possible explanation for the increased chromosomal translocations observed in mammary epithelial cells transformed *in vitro* by aluminium (36) and indicate that a clastogenic effect might contribute to mammary epithelial cell transformation by this metal. These findings were confirmed and extended in experiments using V79 hamster lung fibroblasts, listed in the Organisation for Economic Co-operation and Development (OECD) protocols for the assessment of chemical carcinogens (40). When cultured in the presence of aluminium chloride in the 10μM-1mM range, V79 cells rapidly (within 1 hour) incorporate the metal and by 24 hours incubation exhibit an increase in DSB and an alteration of chromosome numbers (aneuploidy) in metaphase preparations. These effects were dose-dependent and based on large samples (approximately 300 metaphases/sample) and accurate statistics (41). Importantly, at the same doses, aluminium had little cytotoxicity (36, 41). Therefore, mammalian cells exposed to aluminium have the potential to propagate chromosome abnormalities, thus generating the genomic heterogeneity known to underlie tumor formation and progression.

## Discussion

Following our reports on mammary epithelial cell transformation by chronic exposure to aluminium (37, 38) that first highlighted the carcinogenic potential of this metal, our new results on aluminium-induced chromosomal instability observed as an early effect in the context of cellular transformation experiments raise the point on the implication of aluminium in breast carcinogenesis again, and with additional arguments. First, they provide one likely mechanism for the transforming effects of aluminium. Second, like cellular transformation, chromosomal instability occurred in cells exposed to aluminium concentrations close to those measured in the mammary gland of women living in industrialized countries (3). Third, our results in mammary epithelial cells were confirmed and extended in V79 cells, a well-recognized model for the assessment of chemical carcinogens in human toxicology (41). Aluminium is currently not classified as a carcinogenic, mutagenic, or toxic for reproduction (CMR) substance (42). Our results plead in favour of a reassessment.

Recently mandated by the European Union to assess the hazardousness of aluminium salts in the framework of the Registration, Evaluation, Authorization and Restriction of Chemicals (Reach) Regulation, French expert toxicologists of the *Agence nationale de sécurité sanitaire, de l’alimentation, de l’environnement et du travail* (ANSES) identified the possible risk that these compounds induce DNA damage and are potentially mutagenic. According to the current regulatory procedures (it is up to the manufacturers to demonstrate the safety of their own products) they asked the aluminium industry to perform *in vivo* experiments (combined *in vivo* mammalian erythrocyte micronucleus test in bone marrow and modified *in vivo* mammalian comet assay on the liver, kidney, glandular stomach and duodenum; test methods OECD 474 and OECD 489 in rats, oral route, using the registered substance aluminium sulphate) (43) to better evaluate this point. Their request, validated by the other Member States and endorsed by the European Chemicals Agency (ECHA), was rejected by the industry. Seized by the latter, ECHA’s board of Appeal agreed with them on a juridic basis (44). An existing study (45) where a related salt (aluminium acetate) increases micronuclei in the bone marrow of Swiss albino mice following intraperitoneal injection adds interest to the experiments requested by the ANSES.

Looking back to the history of cancer risk factors, hypotheses that have been grossly dismissed or underestimated at the time they arose are now scientifically and clinically established facts, the most notorious example being asbestos (46). In our view, we might have one such story with aluminium. Whereas prospective epidemiological studies comparing (breast) cancer incidence in aluminium-exposed *vs* non exposed series, as well as additional investigations on the potential mutagenic effects of aluminium and their underlying mechanisms remain awaited, the data accumulated so far are sufficient, in our view, to consider restricting the use of aluminium in those situations where absorption is susceptible to occur, based on the precautionary principle.

## Data availability statement

The original contributions presented in this study are included as references. Further inquiries can be directed to the corresponding author/s.

## Author contributions

SJM wrote the first version of this article. This was later edited according to APS comments and submitted for publication. Both authors have made a substantial, direct, and intellectual contribution to the work and approved it for publication.
